# Overexpression of RhoH Permits to Bypass the Pre-TCR Checkpoint

**DOI:** 10.1371/journal.pone.0131047

**Published:** 2015-06-26

**Authors:** Norimasa Tamehiro, Hiroyo Oda, Mutsunori Shirai, Harumi Suzuki

**Affiliations:** 1 Department of Immunology and Pathology, Research Institute, National Center for Global Health and Medicine, Chiba, Japan; 2 Department of Microbiology, Yamaguchi University School of Medicine, Ube, Japan; INSERM-Université Paris-Sud, FRANCE

## Abstract

RhoH, an atypical small Rho-family GTPase, critically regulates thymocyte differentiation through the coordinated interaction with Lck and Zap70. Therefore, RhoH deficiency causes defective T cell development, leading to a paucity of mature T cells. Since there has been no gain-of-function study on RhoH before, we decided to take a transgenic approach to assess how the overexpression of RhoH affects the development of T cells. Although RhoH transgenic (RhoH^tg^) mice expressed three times more RhoH protein than wild-type mice, β-selection, positive, and negative selection in the thymus from RhoH^tg^ mice were unaltered. However, transgenic introduction of RhoH into Rag2 deficient mice resulted in the generation of CD4^+^CD8^+^ (DP) thymocytes, indicating that overexpression of RhoH could bypass β-selection without TCRβ gene rearrangement. This was confirmed by the in vitro development of DP cells from Rag2^-/-^RhoH^tg^ DN3 cells on TSt-4/Dll-1 stroma in an Lck dependent manner. Collectively, our results indicate that an excess amount of RhoH is able to initiate pre-TCR signaling in the absence of pre-TCR complexes.

## Introduction

T cells develop in the thymus through a complex multistage process. It is well known that two major checkpoints exist during T cell development in the thymus. The first checkpoint is β-selection (or pre-TCR checkpoint) at the CD4^-^CD8^-^CD25^hi^CD44^low^ (DN3) stage, and the other is repertoire selection (positive and negative selection) at the CD4^+^CD8^+^ double positive (DP) stage. Progression through both stages is dependent on pre-TCR or TCR complexes, therefore many TCR signal-related molecules are involved in these checkpoints [1, 2].

RhoH belongs to the Rho family small GTPases, which play crucial roles in the development of thymocytes [3]. RhoH is an atypical small G protein that lacks GTPase activity, and is expressed exclusively in hematopoietic lineage cells [4, 5]. We and others have demonstrated that RhoH acts as an adaptor protein associating with the Src-family protein tyrosine kinase Lck, c-Src tyrosine kinase Csk, and Syk-family protein tyrosine kinase Zap70 and Syk [6–8]. Because RhoH is anchored to the plasma membrane through myristoylation, it is able to recruit Lck, ZAP70, and Syk to the membrane to facilitate membrane proximal signal transduction [6, 7, 9]. Ligation of TCR by MHC/peptide complexes induces phosphorylation of TCR-ζ chain ITAMs by Lck, thereby initiating TCR-dependent signal transduction. Zap70 is recruited to these phosphorylated Immunoreceptor tyrosine-based activation motifs (ITAMs), where Zap70 is phosphorylated and converts to the catalytically active form that phosphorylates SLP76 and LAT which then transduce signals to their downstream targets. Since RhoH controls these two proximal kinases important for the initiation of TCR signaling, it is crucial for T cell development and activation. Consequently, TCR-mediated signal transduction in RhoH deficient T cells is almost completely abolished, resulting in defective T cell development through impaired β-selection, positive selection, and negative selection [4, 5, 10].

β-selection, one of the two major checkpoints during T cell development, ensures successful production of a TCRβ chain. Therefore, mice having defective gene rearrangement, such as Rag1/2 deficiency or *scid* mutation, could not pass beyond the checkpoint, resulting in a strict developmental arrest at the DN3 stage. However, the following treatments have been shown to allow checkpoint bypass without TCRβ protein synthesis: irradiation-induced DNA damage [11, 12], inhibition of apoptosis [13, 14], or forced activation of pre-TCR signaling [15–17]. Indeed, constitutively active mutations in signaling molecules such as Lck, Ras and Rac could also induce DN-DP transition independent of TCRβ gene rearrangement [16, 18, 19].

Besides a gene knockout approach, gain-of-function analysis, as typified by transgenic overexpression, is also useful to explore gene function. In this article, we have investigated the function of RhoH by utilizing RhoH transgenic (RhoH^tg^) mice under the control of the CD2 promoter. Overexpression of RhoH in thymocytes did not interfere with T cell development significantly, however, we found that excess RhoH resulted in bypass of the β-selection checkpoint, allowing differentiation from DN to DP without TCRβ recombination *in vivo*. This DN to DP transition was also observed by *in vitro* culture, and was dependent on Lck. Since RhoH facilitates activation of Lck and Zap70, excess amounts of RhoH protein could initiate Lck-dependent activation of pre-TCR signaling in the absence of pre-TCR complexes.

## Materials and Methods

### Ethics statements

Animal experiments were approved by the Animal Care and Use Committee of the National Center for Global Health Medicine (NCGM) Research Institute and conducted in accordance with institutional procedures. (#14033) All efforts were made to minimize suffering.

### Animals

RhoH^tg^ mice were generated by the microinjection of VA hCD2-HA-RhoH vector into the pronuclei of fertilized eggs using standard procedures. Previous studies have shown VA vector containing human CD2 promoter/T cell specific transcriptional enhancer [20] directs the expression of transgenes in mice to the T cell lineage [21]. The embryos were transferred to the oviducts of pseudopregnant ICR female mice. Established mouse lines were maintained after 10 generations of backcrossing to C57BL/6J (B6), and housed under specific pathogen-free conditions in accordance with institutional guidelines. Mice were sacrificed by cervical dislocation to dissect organs out.

### Reagents and antibodies

Rat monoclonal (3F10) and mouse monoclonal antibodies to HA were purchased from Roche (Indianapolis, IN) and Recenttec (Taipei, Taiwan), respectively. Anti Phospho-Src family (Tyr416, #2101S) and Phospho-ERK1/2 (T202/Y204, 197G2) were purchased from Cell signaling (Danvers,MA). RhoH mouse monoclonal antibody (3D3) was purchased from Novus Biologicals (Littleton CO). Monoclonal antibodies to CD2 (RM2-5, 2 μg ml^-1^), CD4 (GK1.5, 0.5 μg ml^-1^), CD5 (53–7.3, 2 μg ml^-1^), CD25 (PC61.5, 2 μg ml^-1^), CD44 (IM7, 2 μg ml^-1^), CD62L (MEL-14, 2 μg ml^-1^), NK1.1 (PK136, 2 μg ml^-1^), TCRb (H57-597, 2 μg ml^-1^), TCRgd (GL3, 2 μg ml^-1^) and Ter-119 (TER-119, 2 μg ml^-1^), and control antibodies for Armenian hamster IgG and rat IgG were purchased from eBioscience (San Diego, CA). These antibodies or reagents were directly coupled to allophycocyanin (APC), APC/cyanine 7 (Cy7), Brilliant Violet 421, fluorescein isothiocyanate (FITC), phycoerythrin (PE), PE/Cy7, or biotin.

Recombinant murine interleukin (IL) -7 was purchased from R&D Systems (Minneapolis, MN). Lck inhibitor III was purchased from CALBIOCHEM (La Jolla, CA).

### Flow cytometry

To obtain single cell suspensions, lymphoid tissues from 4 to 8 week old mice were crushed and passed through 42 μm pore nylon mesh. 1x10^6^ cells were stained with saturating concentrations of indicated antibodies for 30 min at 4°C, washed in 200 μl of staining medium (HBSS containing 0.1% BSA and 0.1% NaN_3_) for 3 times. If need be, cells were then stained with saturating concentrations of appropriate fluorochrome conjugated streptavidin or Goat-F(ab’)2 anti-rabbit IgG for 10 min at 4°C. Cells were analyzed on FACSCanto II (Becton Dickinson, San Jose, CA). For cell sorting of DN3 cells, isolated cells were stained and sorted by fluorescent cell sorting system FACS Aria III (Becton Dickinson, San Jose, CA).

### In vitro T cell differentiation

5x10^5^ cells were cultured with TSt-4/Dll-1 stromal cells [22], which were retrovirally transduced with the murine *Dll-1* gene, for 14 days in 6 well plates. Cells were maintained with RPMI 1640 medium (Sigma) supplemented with 10% (v/v) FCS, L-glutamine (2 mM), sodium pyruvate (1 mM), sodium bicarbonate (2 mg ml^-1^), non-essential amino acid solution (0.1 mM) (Gibco BRL), 2-mercaptoethanol (50 μM), streptomycin (100 mg ml^-1^), penicillin (100 U ml^-1^), and IL-7 (0.5 ng ml^-1^).

### Statistical analysis

Statistical analyses were performed using GraphPad Prism statistical analysis software. Group differences were analyzed by unpaired Student's *t*-test or two-way ANOVA with multiple comparisons, followed by Tukey’s post-test comparisons, for three or more groups. The *p* values ≤ 0.05 were considered significant.

## Results

### HA-tagged RhoH transgene restored normal thymocyte differentiation in RhoH deficient mice

**Fig 1:**
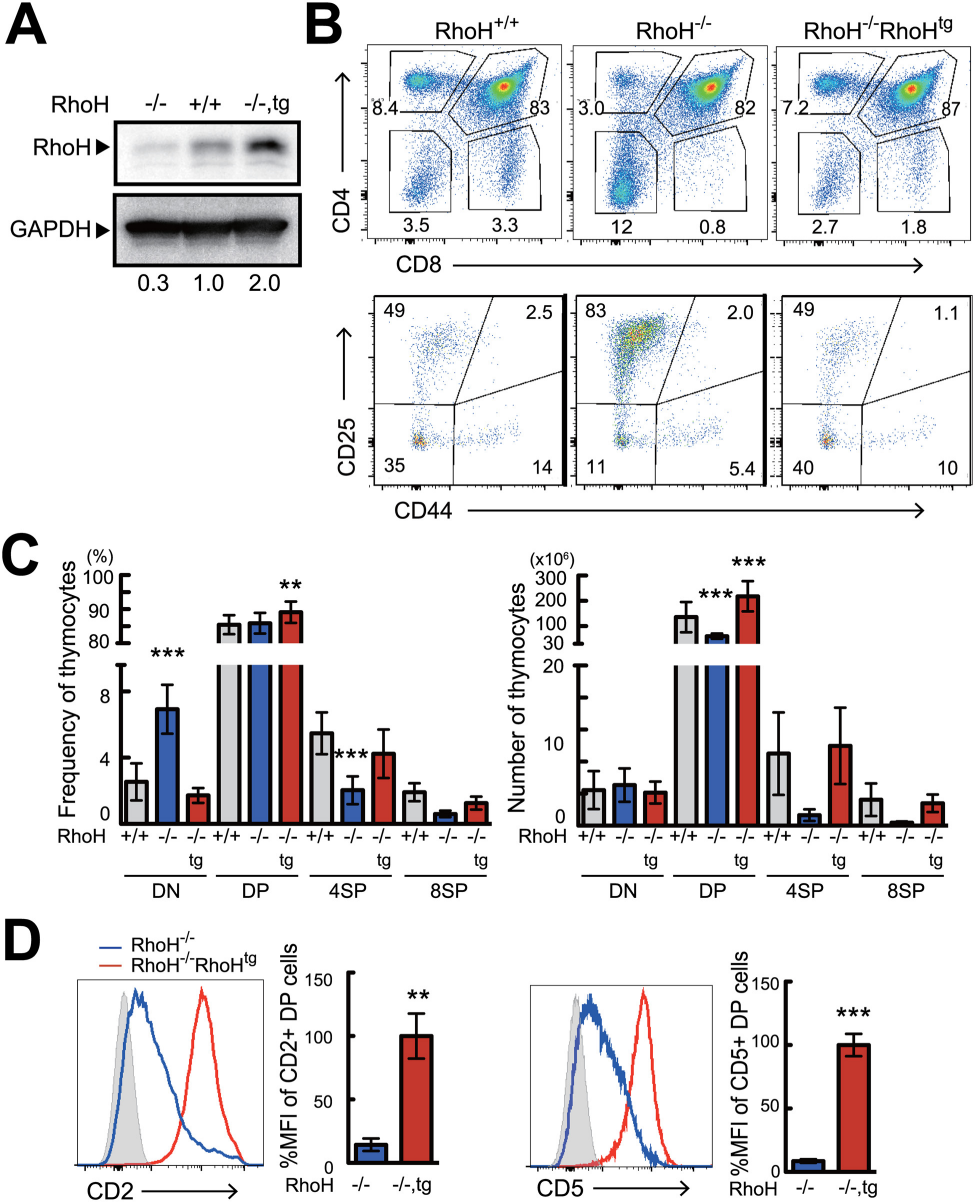
The transgenically expressed HA-tagged RhoH is capable of compensating T cell development in RhoH^-/-^ mice. (A) Analysis of RhoH protein expression by western blot in RhoH^-/-^, RhoH^-/-^RhoH^Tg^, and RhoH^+/+^ thymocytes. (B, C) Analysis of RhoH^-/-^, RhoH^-/-^RhoH^Tg^, and RhoH^+/+^ thymocytes by flow cytometry. Two parameter plots show CD4 versus CD8 surface staining of thymocytes (upper), and CD25 versus CD44 surface staining on CD4^-^CD8^-^ (DN) cells (lower). Numbers indicate percentage of cells in the selected area. Bar graphs represent average cell number and frequency of indicated thymocyte subsets calculated from six mice per group. (D) Single parameter histogram plots show CD2 and CD5 staining in DP thymocytes gated on CD4^+^CD8^+^ cells (n = 6). Data are shown as mean +SD of more than four mice representative of independent experiments. *P<0.05, **P<0.01, ***P<0.001

We established hCD2 promoter-driven RhoH^tg^ mice that expressed an N-terminal HA-tagged RhoH protein. To confirm whether HA-tagged RhoH protein could function normally, RhoH^tg^ mice were bred to RhoH^-/-^ mice. In the RhoH^-/-^RhoH^tg^ mice, protein expression of RhoH in total thymocytes was increased two-fold compared to wild-type mice (Fig 1A). Like wild-type non-tagged RhoH protein, most of the HA-tagged RhoH protein in RhoH^tg^ thymocytes localized to the plasma membrane (S1A Fig). As has been published before, RhoH deficient mice showed severe inhibition of T cell development (Fig 1B and 1C) with thymic cellularity and the percentage of CD4SP and CD8SP cells being significantly reduced [4, 5]. We found that introduction of an HA-tagged RhoH transgene into RhoH^-/-^ mice successfully restored cellularity and the differentiation of CD4SP and CD8SP cells (Fig 1B and 1C). The expression of the transgene were able to restore both defective positive and β-selection (Fig 1B). Expression of CD2 and CD5 on DP thymocytes, which was severely reduced in RhoH^-/-^ [4, 5], was restored by the transgene as well (Fig 1D). The HA-tagged RhoH associated with phosphorylated Lck without stimulation, and was phosphorylated and bound to ZAP70 upon TCR-stimulation (S1B Fig), exactly the same as non-tagged endogenous RhoH. Furthermore, impaired TCR-induced phosphorylation of ERK in RhoH^-/-^ thymocytes was restored by the HA-tagged RhoH transgene (S1C Fig). Collectively, these results demonstrate that HA-tagged RhoH is functionally equivalent to the endogenous one, indicating that HA-RhoH transgenic mice are a proper model for analyzing the molecular functions of RhoH.

### Effects of over-expression of RhoH on T cells in the thymus and peripheral tissues

**Fig 2:**
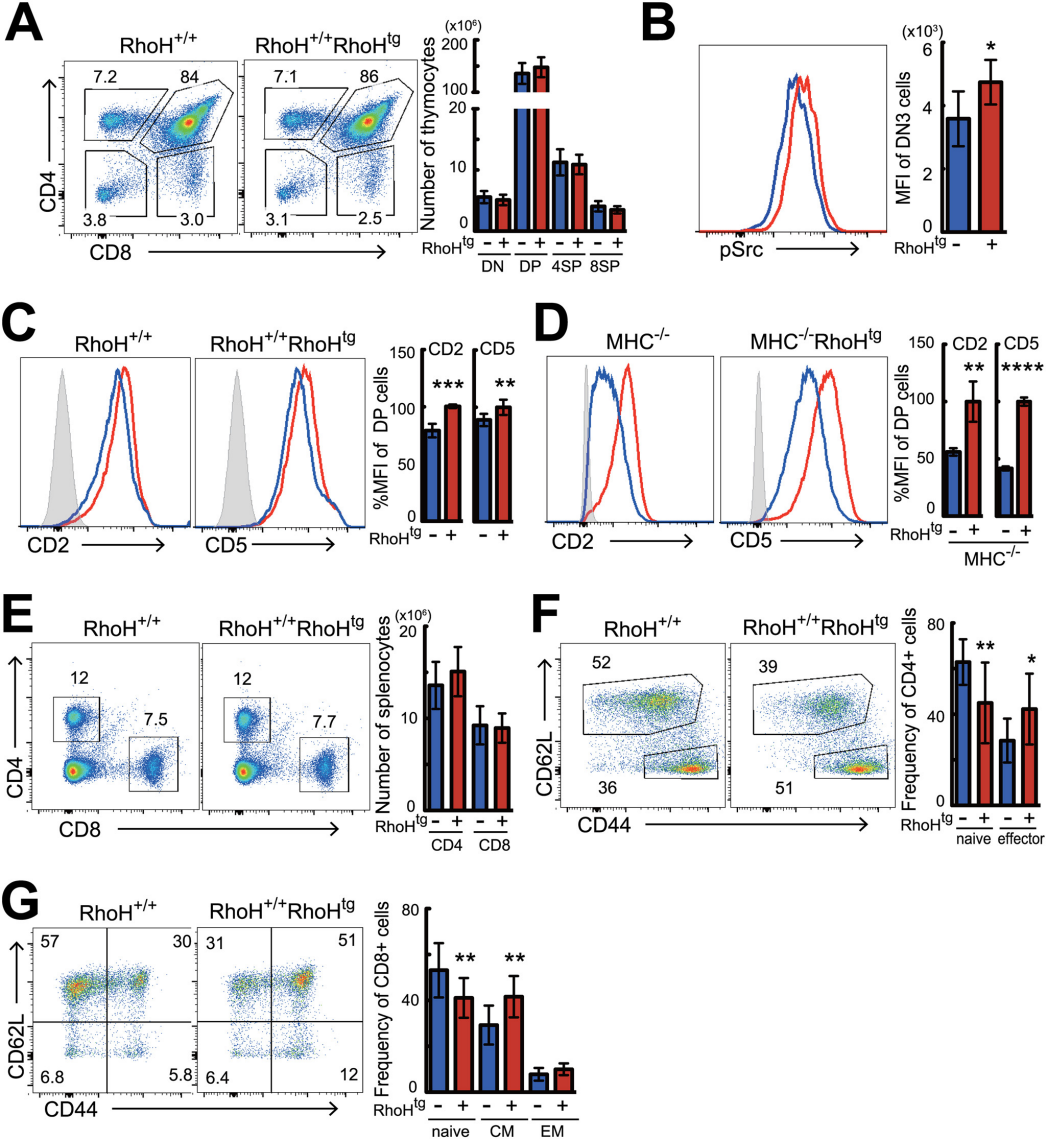
Analysis of T cell development in RhoH overexpressing mice. Analysis by flow cytometry of thymocytes and splenocytes from RhoH^+/+^RhoH^Tg^ (red) and RhoH^+/+^ (blue) mice. Representative two parameter plots show CD4 versus CD8 staining on thymocytes (A, n = 8) and splenocytes (E, n = 13). (B) Representative single-parameter histogram plots show intracellular staining of Phospho-src (pY416) gated on CD4^-^CD8^-^CD44^-^CD25^+^ (DN3) cells (n = 5). Representative single-parameter histogram plots show cell surface staining of CD2 and CD5 antigens on DP cells from RhoH^+/+^RhoH^Tg^ and RhoH^+/+^ mice in either MHC^+/+^ (C, n = 6) or MHC^-/-^ (D, n = 5) background. Solid line and dashed line represent RhoH^+/+^ and RhoH^+/+^RhoH^Tg^, respectively. (F, G) Flow cytometric analysis of CD44 versus CD62L expression profile on splenic CD4^+^ T (F) or CD8^+^ T (G) cells gated on TCRβ^+^ T cells (n = 10). Data are shown as mean +SD and samples were from more than four independent experiments. **P<0.01, ***P<0.001, ****P<0.0001.

To determine gain-of-function effects of RhoH on T cell development, we examined HA-tagged RhoH^tg^ mice in C57BL/6 background. Although lack of RhoH caused strong inhibition of T cell development [4, 5] overexpression of RhoH did not affect overall differentiation of T cells, as the number and proportion of each subset in the thymus were unchanged (Fig 2A and S2A Fig). GFP expression on DP to CD4SP cells from RhoH^tg^ Nur77-GFP mice, which were able to monitor the strength of TCR signaling *in vivo* during T cell developments [23], was also the same as the wild-type controls (S3A Fig). We finally observed RhoH transgene made small increase of the phosphorylation of Src family protein which act as the key kinases on T cell development in thymus (Fig 2B), but subpopulations of DN thymocytes were all unaltered in RhoH^tg^ mice (S2C Fig), indicating that β-selection was not affected. Although prominent differences were not observed in the thymus, we noticed a slight but consistent up-regulation of CD2 and CD5 expression in DP cells from RhoH^tg^ mice (Fig 2C). These changes were still observed in MHC^-/-^ (I-Aβ and β_2_m DKO) background (Fig 2D), which is defective in positive and negative selection, indicating that the increase of CD2 and CD5 was independent of any events later than positive selection. Since expression levels of CD2 and CD5 correlate well to the strength of TCR signal [24, 25], these results may indicate augmented pre-TCR signaling in RhoH-overexpressing thymocytes. In the periphery, the percentage and numbers of splenic CD4^+^ and CD8^+^ T cells from RhoH^tg^ mice were similar to that of wild-type mice (Fig 2E and S2B Fig). However, frequency and numbers of naïve CD4 and CD8 T cells (CD44^low^CD62L^hi^) were significantly reduced, and activated/memory T cells were concomitantly increased in RhoH^tg^ mice (Fig 2F and 2G). Collectively, compared to the severe phenotypes of RhoH deficient mice, overexpression of RhoH had little effect on T cells, apart from the increased phosphorylation of Src family kinases in DN3, increased expression of CD2 and CD5 on DP, decreased number of naive T cells, and increased activated/memory T cells in the periphery. Other than conventional TCRαβ lineage T cells, development of unconventional T cells such as pTregs, NKT cells, and TCRγδ T cells was not changed in the RhoH^tg^ mice (S4A–S4C Fig).

### Overexpression of RhoH enables bypass of β-selection in Rag2^-/-^ mice

**Fig 3:**
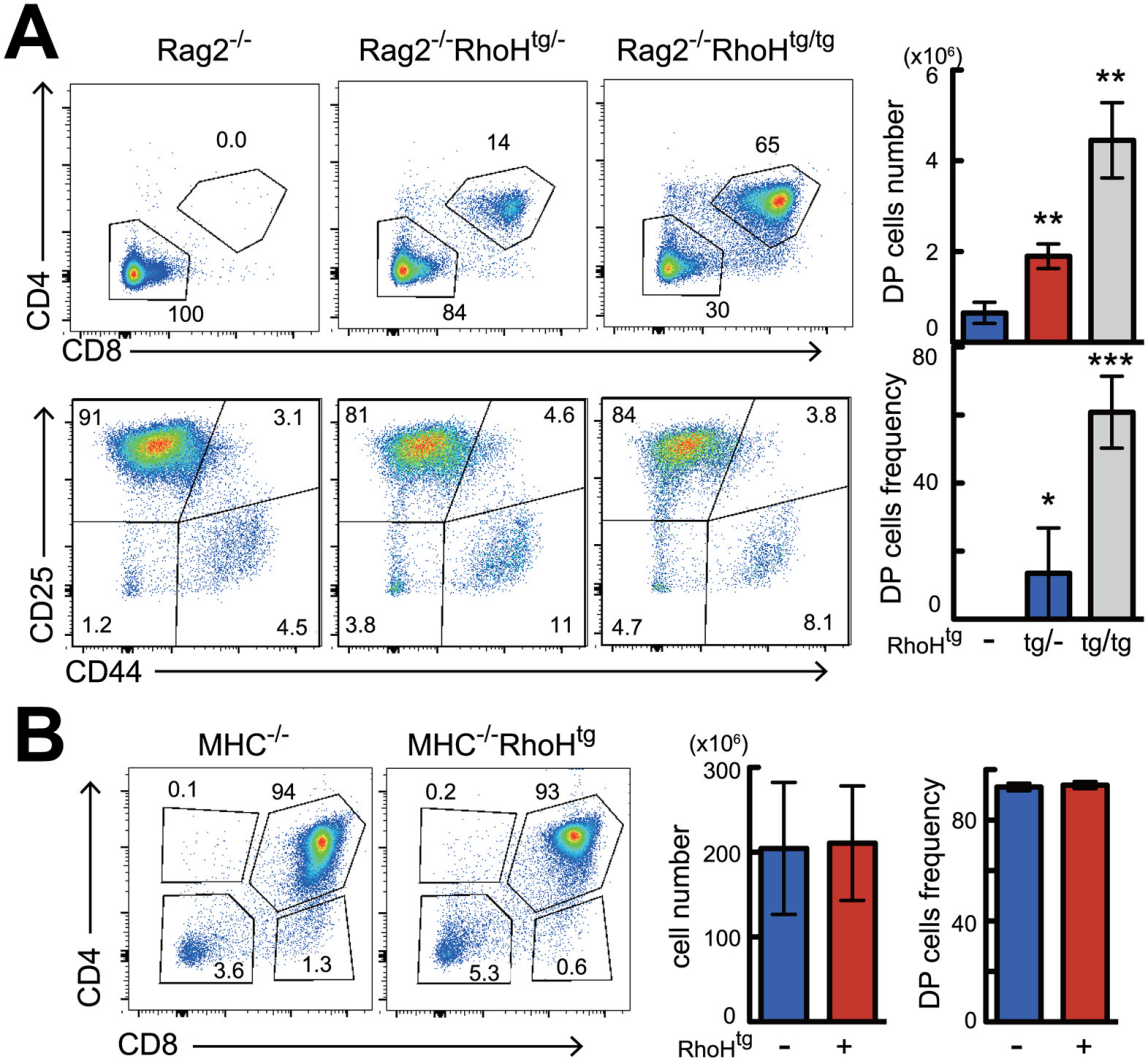
RhoH transgene expression enables transition from DN to DP stages in Rag2^-/-^ mice. FACS analysis of thymocyte differentiation in Rag2^-/-^ (A) or MHC^-/-^ (B) background. Representative two parameter plots show CD4 versus CD8 staining on thymocytes and CD25 versus CD44 staining on DN cells. Bar graphs represent average cell number and frequency of indicated thymocyte subsets calculated from five mice per group. **P<0.01, ***P<0.001.

Rag2 deficient mice are unable to complete gene rearrangement of the TCR locus, therefore T cell development stops at the β-selection checkpoint, resulting in complete arrest at the DN3 stage. To our surprise, when we crossed RhoH^tg^ mice with Rag2^-/-^ mice, DP thymocytes emerged in the thymus (Fig 3A). The generation of DP cells associated with increased gene expression of RhoH was in a dose-dependent manner (Fig 3A), because transgene homozygous tg/tg mice generated more DP than transgene heterozygous tg/- mice. Introduction of RhoH transgene did not induce gene rearrangement in the TCRβ locus, because TCRβ protein was not observed in these DP cells (S5A Fig). Overexpression of RhoH could successfully bypass β-selection to generate DPs, however it could not bypass positive selection because differentiation of mature CD4SP and CD8SP cells was not observed in the Rag2^-/-^RhoH^tg^ mice (Fig 3A). This was further confirmed by the results from MHC^-/-^RhoH^tg^ mice, which contained no single positive cells (Fig 3B), proving that simple overexpression of RhoH is not sufficient for bypassing positive selection. Taken together, overexpression of RhoH enabled bypass of β-selection without TCRβ-gene rearrangement, however it was not sufficient to permit bypass of positive selection.

### RhoH-induced bypass of β-selection is Lck-dependent

**Fig 4:**
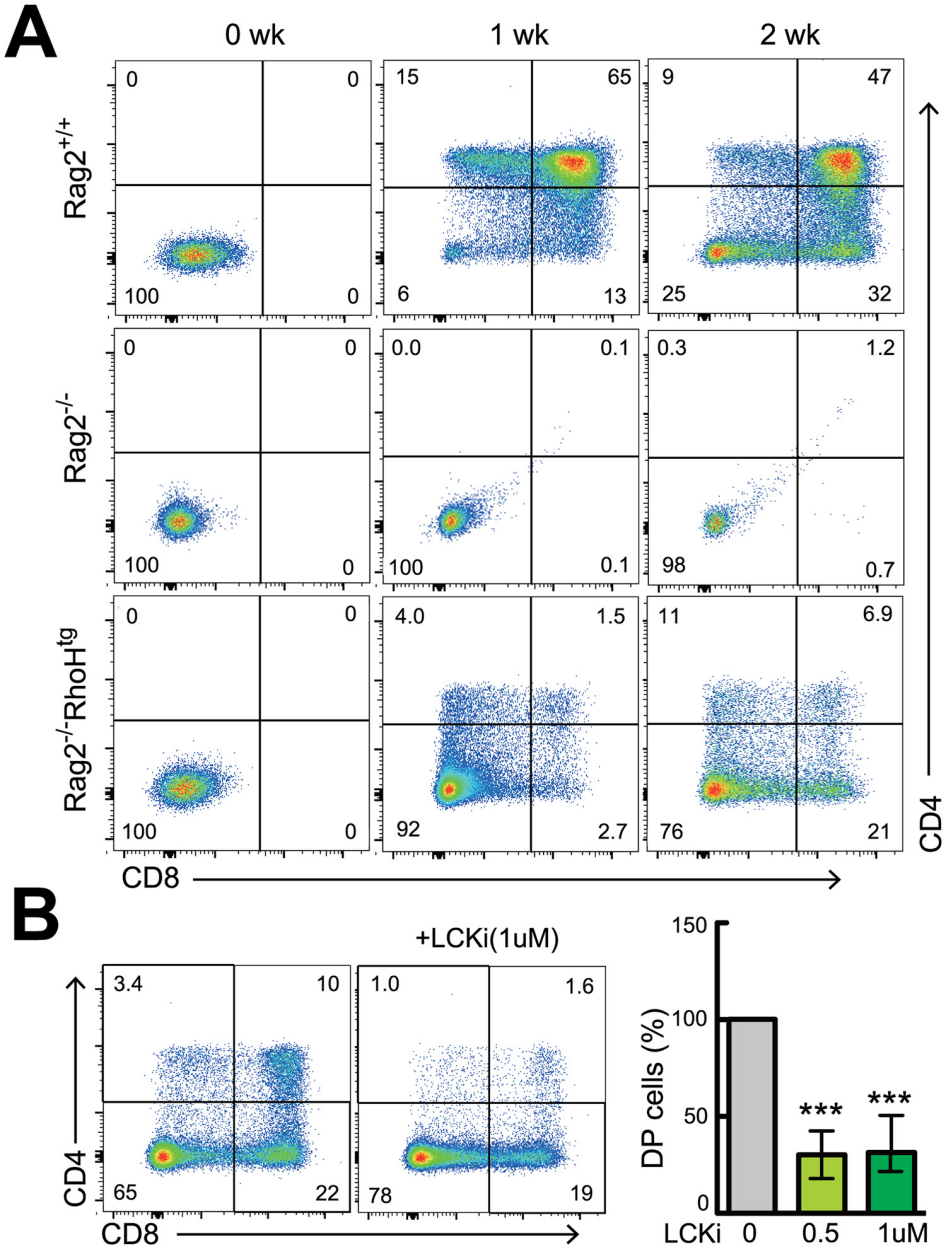
Lck activation is required for RhoH transgene-induced DPs generation without TCRβ-gene rearrangement. (A) FACS analysis of the *in vitro* differentiation of thymocytes to the DP stage. DN3 cells from Rag2^-/-^, Rag2^-/-^RhoH^tg^, and Rag2^+/+^ C57B6 mice were differentiated for 14 days with the stromal cell line TSt-4/Dll-1 in the presence of IL-7. Representative two parameter plots show CD4 versus CD8 staining on cultured thymocytes at the indicated days. Results shown are from one out of three independent experiments. (B) DN3 cells were cultured with vehicle alone or a pharmacological inhibitor of Lck at the indicated concentration. Bar graphs represent the percentages of DP thymocytes compared with the vehicle control group. Data are representative of more than three independent experiments. ***P<0.001.

To address whether generation of DPs in Rag^-/-^RhoH^tg^ mice was thymocyte intrinsic, we next tested using an *in vitro* differentiation culture system. Sorted DN3 cells from wild-type mice were able to differentiate into DPs *in vitro* in a week on monolayers of Notch ligand-expressing stromal cells (TSt-4/Dll-1), while DN3 cells from Rag2^-/-^ mice could not (Fig 4A). We found that sorted DN3 cells from Rag2^-/-^RhoH^tg^ mice could successfully differentiate into DP cells (Fig 4A), demonstrating that these DPs were truly differentiated from DN3 cells, and that this transition was thymocyte intrinsic. Furthermore, this *in vitro* DPs generation was strongly inhibited by the addition of Lck inhibitor (Fig 4B, S6 Fig), indicating that RhoH-transgene induced generation of DPs was dependent on Lck activity.

## Discussion

The current report describes, for the first time, the effect of RhoH-overexpression on the development and activation of T cells. Overexpression of RhoH had little impact on T cell development, although lack of the molecule induces severe defects [4, 5]. As a matter of fact, the only differences we observed were the slight increase of phospho-Src in DN3, increase of CD2 and CD5 expression on DP thymocytes, and reduction of naive T cells in the periphery with a concomitant increase of activated/memory T cells. Other than these differences, the development of T cells was apparently normal.

As has been reported, RhoH acts as an adaptor molecule for Lck and Zap70; kinases important for initiating TCR-dependent signal transduction. Thus, the absence of RhoH causes a severe reduction in TCR signaling. By using RhoH-overexpressing transgenic mice we expected enhanced TCR signal transduction, however, no strong augmentation of TCR-dependent signaling has been observed so far. In addition to the unaltered Nur77-GFP expression during thymic T cell differentiation (S3A Fig), anti-TCR antibody-induced activation of ERK in DP and CD4SP cells from RhoH^tg^ mice was also not increased (S3B Fig), indicating that enhancement of TCR signal transduction was not occurring in DP and CD4-SP from the RhoH^tg^ mice. RhoH is required for proper transduction of TCR-mediated signals; however, the level of RhoH expression in wild-type mice might be sufficient and already saturated regarding the activation of Lck and Zap70. This could explain why the RhoH-overexpressing transgenic mice did not exhibit a strong phenotype.

We observed a slight but consistent increase in CD2 and CD5 expression in DPs from RhoH^tg^ mice (Fig 2C and 2D). Because expression levels of CD2 and CD5 are correlated with the strength of TCR signal [24, 25], this phenotype likely represents increased pre-TCR signaling at the DN3 stage. In fact, we have found that Src family protein including Lck were activated under steady state in DN3 cells from RhoH^tg^ mice, although we did not observe increased TCR-signaling in DP cells (Fig 2B and S3B Fig). Since the expression level of endogenous RhoH protein in DN3 cells is much lower than in DP, the amount of RhoH in DN3 may not be saturated, therefore supplementation with exogenous RhoH expression could enhance pre-TCR signaling.

RhoH has been reported to inhibit Rac activity [26], and overexpression of RhoH into murine hematopoietic progenitor cells via retroviral-mediated gene transfer reduced proliferation and chemotaxis, and increased apoptosis [27]. However, we did not observe reduced proliferation of CD4-SP (S4D Fig), nor increased apoptosis of DN3 cells (S3C and S5B Figs) in RhoH-tg mice. Therefore function of RhoH seems to be different in different cell types.

We also observed a slight change in TCRab repertoire in RhoH transgenic mice (S4D Fig). This could be explained by inadequate activation of Lck due to RhoH overexpression. It is known that excessive activation of Lck such as in the constitutively active Lck^tg^ mice suppresses allelic exclusion at the TCRβ gene locus [28, 29], resulting in defective T cell development. Therefore, moderate increases in Lck activation may partially disturb allelic exclusion leading to TCR repertoire changes. The other possibility is mediated by IL-7 signaling. IL-7 induces the expression of integrin a_4_b_7_ and preferential expansion of naïve CD4T cells, resulting in diversification of TCR repertoire [30, 31]. Because RhoH deficiency in human revealed relative specific defect in skin homing b_7_ and a_4_b_7_ integrin^+^ T cells [32], further studies are needed to elucidate IL-7 dependency on TCR repertoire change in RhoH^tg^ mice.

We show for the first time that overexpression of RhoH led to bypass of the pre-TCR checkpoint in Rag2^-/-^ mice. RhoH-overexpression did not induce gene rearrangement of the TCRβ locus in Rag2^-/-^ mice, as TCRβ protein was not expressed in DP from the Rag2^-/-^RhoH^tg^ mice (S5A Fig). Bypassing the pre-TCR checkpoint in β-selection-deficient DN cells can be achieved by three independent mechanisms. The first mechanism is associated with DNA strand breaks. DNA damaging agents such as ionizing irradiation-induced p53-dependent [33] generation of DP in SCID mice [11, 34] and Rag2 deficient mice [12, 35]. It is thought that DNA damage induces p53-dependent DNA repair pathways that then somehow induce DP generation. The second mechanism is the inhibition of apoptosis in DN3 cells. The inhibition of apoptosis either by overexpression of FADD-dominant negative protein [13], or inactivation of p53, evoked DP progression in SCID [36], Rag2 deficient [37], and CD3γ deficient [38] thymocytes. DN cells that fail to initiate pre-TCR signaling are destined to die via p53-dependent apoptosis, and inhibition of their death resulted in the unexpected survival of dying cells that could proceed to DP stage. It is of note that p53 functions in a positive way in the first mechanism, whereas it acts in negative way in the second mechanism. The third mechanism is substitution or mimicry of pre-TCR signaling. It was shown that administration of anti-CD3-antibody in Rag2 deficient mice induced DPs [15] by emulating pre-TCR signal transduction. After that, transgenic introduction of various signaling related molecules such as active-Lck [16], CD4 [17], active-Rac [19], RasGRP1 [39], active-Ras [18], CD28/B7 [40], Pim-1 [41], Egr1/NF-ATc1 [42, 43], Egr3 [44], and Ly49D/DAP12 [45] were reported to bypass the pre-TCR checkpoint in Rag2 or CD3ε deficient mice. We found apoptosis are not altered in RhoH over-expressing DN3 cells (S3C and S5B Figs), suggesting that the second mechanism may be not in the case.

RhoH facilitates TCR/pre-TCR signal transduction by the recruitment of Lck and Zap70, therefore overexpression of RhoH would presumably enhance TCR signaling. It could be that the overexpression of RhoH activates Lck in the absence of TCRβ protein, thus generating DP cells by the third mechanism. Indeed, the *in vitro* generation of DPs from RhoH^tg^ DN3 cells on stromal cell monolayers was blocked by Lck inhibitor, indicating that this transition was dependent on Lck, which is consistent with normal differentiation of DN to DP cells. Although we could not observe any apparent increase in anti-TCR mAb-stimulated "strong" signal transduction in RhoH^tg^ mature T cells and DP thymocytes (S3B Fig), it could be different in weaker TCR stimulation, or it could depend on developmental stage. Indeed, the slight increase in CD2 and CD5 on DPs, and the bypassing of β-selection, could be an indication of augmented pre-TCR signaling in DN3 stage. Because expression level of endogenous RhoH was lower in DN3 thymocytes, augmentative effects could be seen in DNs. Therefore, we think the increased expression of RhoH in DN3 initiated Lck-dependent pre-TCR signaling even in the absence of TCRβ protein.

Reduced numbers of naive T cells (CD44^low^CD62L^hi^) of RhoH^tg^ mice implies that RhoH transgene may regulate this subpopulation of peripheral T cells. Because of the paucity of normal mature T cells in RhoH^-/-^ mice, it has been difficult to determine the requirement of RhoH in primary peripheral T cells by using systemic knockout mice. To define the effector function of RhoH in peripheral T cells, further analyses using RhoH conditional knockout mice are needed. In summary, we showed that overexpression of RhoH in DN cells enabled bypass of the β-selection checkpoint during thymocyte development.

## Supporting Information

S1 Fig RhoH^-/-^RhoH^tg^ mice recapitulate major features of normal thymocyte development. (EPS)

S2 Fig Frequency of indicated sub-population in lymphocytes. (EPS)

S3 Fig Monitoring TCR dependent signal transduction in thymocytes. (EPS)

S4 Fig Analysis of the effect of RhoH transgene on peripheral lymphcytes. (EPS)

S5 Fig The effect of RhoH transgene on Rag2-deficient thymocytes. (EPS)

S6 Fig The inhibition of *in vitro* thymocytes differentiation with LCK inhibitor. (EPS)
